# Near-present and future distribution of *Anopheles albimanus* in Mesoamerica and the Caribbean Basin modeled with climate and topographic data

**DOI:** 10.1186/1476-072X-11-13

**Published:** 2012-04-30

**Authors:** Douglas O Fuller, Martha L Ahumada, Martha L Quiñones, Sócrates Herrera, John C Beier

**Affiliations:** 1 Department of Geography and Regional Studies, University of Miami, Miami, FL, USA; 2 Entomology Group, Instituto Nacional de Salud, Bogotá, Colombia; 3 Public Health Department, Faculty of Medicine, National University of Colombia, Ciudad Universitária, Bogotá, Colombia; 4 Caucaseco Scientific Research Center/Immunology Institute, Universidad del Valle, Cali, Colombia; 5 Department of Epidemiology and Public Health, Miller School of Medicine, University of Miami, Miami, FL, USA

## Abstract

**Background:**

*Anopheles albimanus* is among the most important vectors of human malaria in Mesoamerica and the Caribbean Basin (M-C). Here, we use topographic data and 1950–2000 climate (near present), and future climate (2080) layers obtained from general circulation models (GCMs) to project the probability of the species’ presence, p(***s***), using the species distribution model MaxEnt.

**Results:**

The projected near-present distribution parameterized with 314 presence points related well to the known geographic distribution in the study region. Different model experiments suggest that the range of *An. albimanus* based on near-present climate surfaces covered at least 1.27 million km^2^ in the M-C, although 2080 range was projected to decrease to 1.19 million km^2^. Modeled p(***s***) was generally highest in Mesoamerica where many of the original specimens were collected. MaxEnt projected near-present maximum elevation at 1,937 m whereas 2080 maximum elevation was projected at 2,118 m. 2080 climate scenarios generally showed increased p(***s***) in Mesoamerica, although results varied for northern South America and no major range expansion into the mid-latitudes was projected by 2080.

**Conclusions:**

MaxEnt experiments with near present and future climate data suggest that *An*. *albimanus* is likely to invade high-altitude (>2,000 m) areas by 2080 and therefore place many more people at risk of malaria in the M-C region even though latitudinal range expansion may be limited.

## Background

Malaria is an important vector-borne disease in Latin America and the Caribbean (LAC) with some 775,000 cases reported in 2007 in the region [1]. The majority of cases occur in the Amazon basin, but cases in the non-Amazonian areas of Latin America account for some 40% of the total in LAC [2]. Malaria remains problematic in some countries in Mesoamerica and the Caribbean (M-C) including Haiti (2.8%), Guatemala (3.8%), Honduras (1.5%) and Panama (0.4%) and parts of Mexico (0.3%) [1,2]. Some 90 different anopheline species have been reported in LAC and nine, including *Anopheles albimanus*, are considered dominant malaria vectors [3]. *An*. *albimanus* is also considered one of the major vectors of human plasmodia in Mesoamerica, northern South America and the Caribbean [4]. Recent outbreaks of autochthonous malaria in the Cayman Islands, the Bahamas and Jamaica have been attributed to this vector [5]. *An*. *albimanus* is widespread, and reports indicate that it exploits a diverse range of habitats and is both anthropophilic and zoophilic in its feeding behavior [3]. Moreover, its larvae can tolerate a wide range of water quality conditions, including rice fields, flowing streams, river margins and brackish water associated with mangrove swamps [3]. While the species has normally been found in low-lying areas, usually in locations less than 500 m above sea-level, several reports indicate its presence at higher elevations (up to 1,941 m) [4,6]. Given its apparently broad tolerance limits, its importance in malarial transmission, its broad spatial extent and ample data related to its location, we selected this particular species for our analysis. Moreover, as a generalist species, it may be more likely than more specialist anophelines (e.g., *An. bellator*, which breeds in bromeliads) to invade suitable habitats in mid-latitude or high-altitude locations after decades of atmospheric warming and therefore accurate information on its potential distribution can inform current and future control efforts aimed at limiting its spread.

The distribution limits of many malaria vectors are clearly associated with climate conditions, particularly temperature and precipitation. Temperature ranges are important at each stage of the mosquito lifecycle and influence vectorial capacity [7,8]. Water availability for breeding sites is also fundamental to the survival of malaria vectors and may include, permanent and temporary water bodies, which are generally associated with rainfall timing and amount; and in some anophelines temporary water bodies may provide critical breeding sites in dry environments [9]. Therefore, while temperature can be considered to exert a direct effect on mosquito development, rainfall timing and amount function as indirect environmental factors that condition the environment in ways that affect the fitness of mosquitoes. Elevation functions analogously in that it controls climate conditions at relatively local scales such that elevational gradients in montane environments function much the same as latitudinal gradients, while slope and aspect may be associated with anopheline habitats at local scales within particular landscapes [10]. Moreover, one recent study showed that temperature, rainfall and altitude can explain much of the variance in the distribution of *An. albimanus* in LAC [3].

The mapping of anopheline presence using species distribution models (SDMs) offers many potential advantages over point-based maps that indicate where species have been recorded. While dot maps provide a general indication of where a particular species may be found, SDMs also furnish a means to interpolate between collection points as well as extrapolate outside known bounds. Further, SDMs provide a way to improve disease risk mapping by revealing how environmental changes are likely to affect the future distribution potential of species such as *An*. *albimanus*. Such models typically use geo-referenced collection points in conjunction with environmental covariates that function as predictors to project a potential distribution or probability of presence [11]. To date relatively few studies [12,13] have employed SDMs to ascertain the distribution limits of malaria vectors. Moreover, we are unaware of any published studies that have used SDMs to project future anopheline distributions in the Western Hemisphere based on outputs from general circulation models (GCMs), which predict future temperature and rainfall conditions associated with a range of future socio-economic scenarios [14].

**Figure 1:**
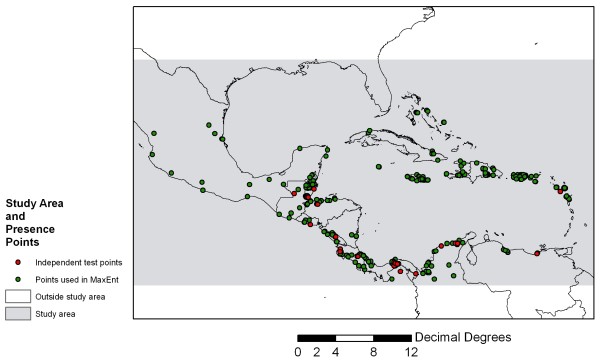
**Distribution of*An. albimanus*records used to parameterize and test MaxEnt projections within the study area.**

In this study, we use mean monthly temperature and rainfall surfaces representing climate conditions during the 1950–2000 period, which we refer to as “near-present” climate and comparable climate data for 2080 derived from three different GCMs, in conjunction with a digital elevation layer and a set of 350 geo-referenced points where *An. albimanus* specimens have been collected in our study area. We used the popular and robust SDM, MaxEnt, to model the species’ near-present and 2080 distributions and we focused on the M-C region (Figure 1) where *An*. *albimanus* is one of nine dominant vectors [3]. Our study addresses three fundamental questions: 1) How does the model perform using climate data to project the near-present distribution of the species? 2) Does the model show that future climate from GCMs will indicate a potential expansion of the vector’s range into the mid-latitudes? 3) How does the model predict elevational shifts in the species’ range with warmer conditions in 2080?

## Results

**Figure 2:**
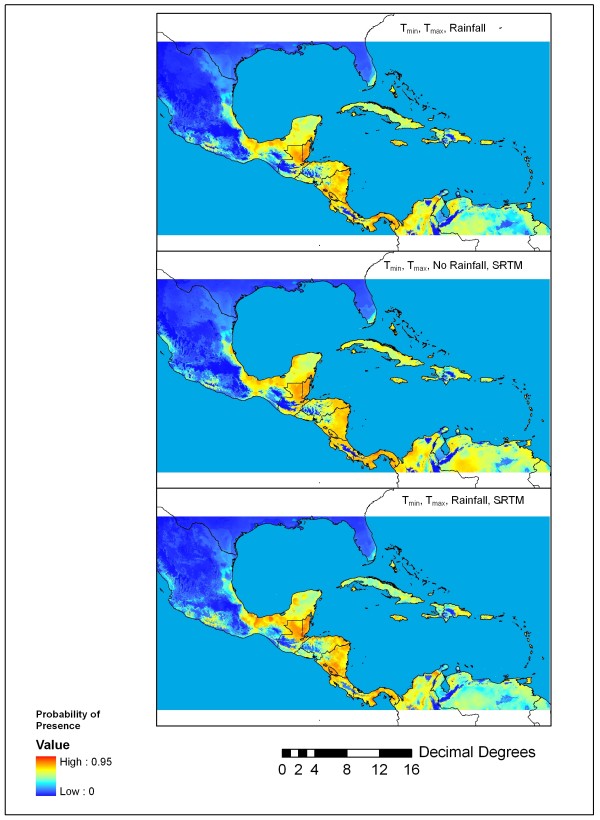
**Probability of*An. albimanus*presence obtained in three different experiments.** Top panel: experiment 56 (monthly T_min_, T_max_, rainfall); middle panel: experiment 58 (monthly T_min_, T_max_, SRTM); bottom panel: experiment 61 (monthly T_min_, T_max_, rainfall and SRTM).

The near-present distribution (Figure 2) from three model experiments (Table 1) matches well with known northern distribution limits, including high probability (>0.50) of presence in Chiapas, Mexico [15], Belize [16], and South Florida [17]. Moreover, the model indicates that the highest probabilities of presence are in the Mesoamerica where many specimens of *An. albimanus* have been collected over the past 60 years (Figure 1). The 36 independent validation or test points indicate a relatively high mean probability values (~0.70) for these maps, while the lowest-presence threshold (LPT) value for experiment 58, in which monthly precipitation was excluded, was low (0.188) suggesting that the model was sensitive to this particular variable. At a threshold of p(***s***) > 0.50, a relatively large omission error of 0.130 in experiment 58 (Table 1) also suggests that precipitation is an important variable to retain for produce accurate projections of *An. albimanus* distribution. It is worth noting that the near-present distribution shown in Figure 2 also indicated high probability of *An*. *albimanus* presence in the southern Caribbean (e.g., Trinidad) where it is considered either absent or secondary in importance to *An. aquasalis*[2,5].

**Table 1:** MaxEnt results for different experiments that yielded relatively high mean probability values (~0.70 or greater) evaluated using 36 independent test points

**MaxEnt Experiment or Crosstabulation (CT)**	**Co‒variates**	**Time**	**Mean (sd)**	**LPT**	**p(*s*) > 0.50 (e**_**o**_**, e**_**c**_**)**	**Elevation (LPT) E**_*max*_**(m)***E*_*mean*_*(m)***(sd)**	**Elevation p(*s*) > 0.50 E**_*max*_**(m)***E*_*mean*_*(m)***(sd)**
56	T_min_, T_max_, PPT	Near present	0.701 (0.100)	0.406	(0.087, 0.000)	1936 *165* (797)	1886 *147* (850)
58	T_min_, T_max_, SRTM	Near present	0.687 (0.143)	0.188	(0.130, 0.000)	2208 *197* (764)	1832 *140* (778)
61	T_min_, T_max_, SRTM, PPT	Near present	0.700 (0.104)	0.427	(0.087, 0.000)	2253 *234* (322)	2253 *239* (319)
68	T_min_, T_max_, SRTM, PPT Handey	2080	0.658 (0.15)	0.076	(0.087, 0.001)	2906 *435* (541)	2258 *267* (346)
69	T_min_, T_max_, SRTM, PPT CSIRO	2080	0.661 (0.103)	0.296	(0.043, 0.001)	2531 *256* (352)	2253 *248* (322)
70	T_min_, T_max_, SRTM, PPT CCCMA	2080	0.678 (0.107)	0.419	(0.087, 0.000)	2174 *222* (302)	2174 *221* (303)
CT Exp 56, 58, 61		Near present				1937 *210* (277)	
CT Exp 68, 69, 70		2080				2118 *231* (307)	

**Figure 3:**
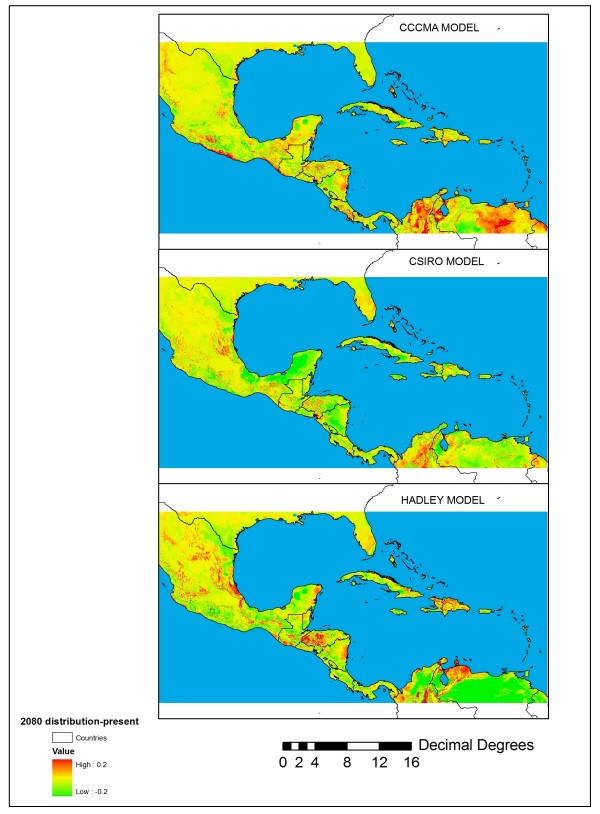
**Differences between near-present probability of presence obtained from experiment 61 and the mode of three different 2080 A2 climates (experiments 68–70).** Top panel: experiment 70 (CCCMA model T_max_, T_min_, rainfall, SRTM) – experiment 61; middle panel: experiment 69 (CSIRO model T_max_, T_min_, rainfall, SRTM) – experiment 61; bottom panel: experiment 68 (Hadley Centre model T_max_, T_min_, rainfall, SRTM) – experiment 61.

Table 1 reveals that the LPT value ranged from relatively low (0.076) for the Hadley Centre model to moderate (0.296) for the CSIRO model to relatively conservative (0.419) for the CCCMA model. Figure 3 shows the difference between experiments 68–70 based on GCM models and near-present distribution shown in experiment 61. This figure reveals large potential differences in the future p(***s***) including wide areas of model disagreement in northern Colombia and Venezuela and some general areas of agreement in Mesoamerica, including places where the three GCM layers suggest increased p(***s***), particularly in the Gulf Coast states of Mexico, as well as parts of Honduras and Nicaragua. The CSIRO model, which produced the most spatially homogeneous predictions for future temperatures, also produced relatively modest changes in p(***s***) relative to the Hadley and CCCMA climate layers. The 2080 Hadley climate (experiment 68), which predicted drier conditions from the Yucatan throughout much of northern South America as well as much warmer conditions relative to the other two GCMs, produced the largest relative p(***s***) decrease in much of northern Venezuela. On the other hand, the CSIRO and CCCMA climates (experiments 69 and 70 respectively), which predicted generally wetter and more moderate temperature increases in 2080 resulted in elevated p(***s***) values in parts of northern Colombia, eastern Nicaragua, and Honduras. Overall, the results suggest that relatively high projected temperature increases (6°C or greater) by 2080 coupled with large negative departures in mean annual rainfall will cause MaxEnt to project decreased p(***s***) in the M-C region.

**Figure 4:**
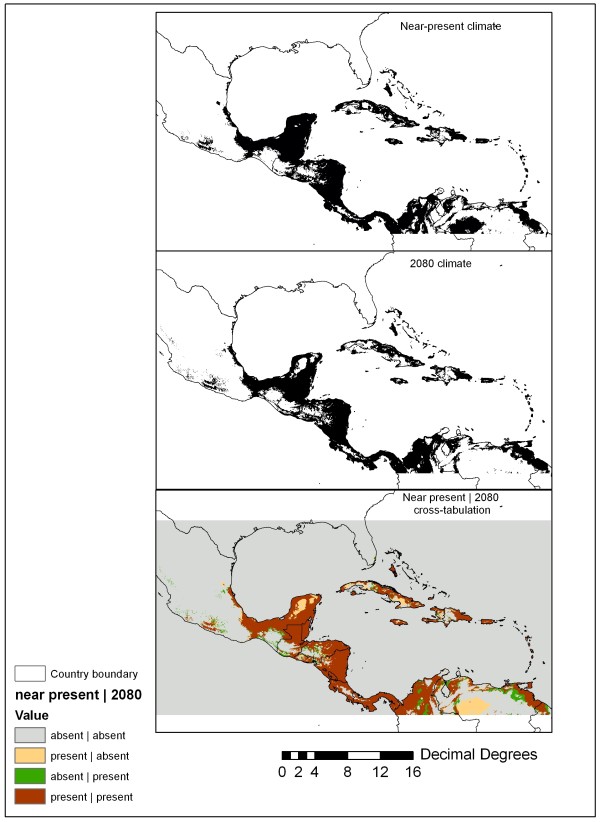
**Least-presence threshold applied to the mode of experiments 56, 58, and 61 (top panel) and the mode of experiments 68–70 (middle panel).** The bottom panel shows the cross-tabulation of the top and middle panels to reveal how the range of *An. albimanus* may shift from near present climate conditions to 2080.

Figure 4 shows the projected distribution based on the lowest-presence threshold (Table 1) applied to near-present (mode of experiments 56, 58, 61) and 2080 climates (mode of experiments 68–70). This figure also shows the full cross-tabulation of near present and 2080 distributions (bottom panel) as well as potential changes in *An*. *albimanus* range with future climate change. Areas of range contraction include portions of southeast and western Cuba, the Yucatan Peninsula, and northern Venezuela. Some projected range expansion can be seen along the margins of the existing range in northeast Venezuela, the interior of northern Colombia, and southern Mexico. Overall, however, Figure 4 does not suggest a major expansion of *An*. *albimanus* into the mid-latitudes except for a small area of expansion in south Florida. Different model experiments suggest that the range of *An. albimanus* based on near-present climate surfaces covered at least 1.27 M km^2^ in the M-C, although by 2080 the range is projected to decrease to 1.19 M km^2^. While MaxEnt did not predict major latitudinal shifts by 2080, the model experiments indicated major potential for upslope colonization, particularly in the case of the Hadley climate, which caused MaxEnt to project a maximum elevation of 2,906 m in 2080. In contrast, the more conservative CCCMA and CSIRO climates produced projected elevation maxima (E_max_) of 2,174 m and 2,531 m, respectively (2080 E_max_ range = 732 m). Comparing the areas of agreement in near-present and future climates obtained through cross-tabulation (CT in Table 1), the model experiments suggest that E_max_ will increase from 1,937 m to 2,118 m from near-present to 2080 and that mean elevation (E_mean_) will increase from 210 m to 231 m over the same period. With the higher threshold of p(***s***) > 0.50 applied, the predicted E_max_ and E_mean_ values were more conservative; for example, at p(**s**) > 0.50, the E_max_ range was 421 m and 84 m for the near-present and 2080 climates, respectively, with most of the difference attributable to the different thresholds applied to the Hadley climate experiment.

## Discussion

Our model experiments with MaxEnt provide new insights into how the distribution of *An. albimanus* may change by 2080 as a result of climate change. The projected areas of range expansion appeared to be spatially coherent and proximate to projected areas of near-present range, which is to be expected. Although we expected that MaxEnt would project a northern expansion of *An. albimanus* range into parts of the Gulf Coastal region and South Florida by 2080 with warmer conditions, the model experiments for the most part did not reveal this. The lack of predicted range expansion is at odds with a study by Rogers and Randolph [18], which suggested an increased area of malaria potential in the southern United States by 2050 as a result of climate change. Of course, many other vectors may be implicated in future malaria outbreaks in the mid-latitude portions of our study area including dominant vectors such as *An*. *aquasalisAn*. *quadrimaculatusAn. pseudopunctipennisAn. darlingiAn.vestitipennis, An. nuneztovari,* which are all reported to be present in Central and northern South America [3,8].

Consistent with general understanding of how climate relates to mosquito fitness, future climate changes that involve large increases in temperature (e.g., > 6°C) and much drier conditions tended to result in lower p(***s***). A small projected range contraction of approximately 80,000 km^2^ (or about 6.3%) suggests that overall climate conditions will become more unfavorable to *An. albimanus* in certain parts of the region. We interpret the potential decrease in p(***s***) as a function of drier and warmer conditions in parts of northern South America and Central America associated with El Niño events [19], which several GCM experiments suggest may become more frequent by the end of this century [14]. The potential highland expansion of *An. albimanus* by 2080 was also expected and consistent with one other study that showed that populations of this species are encroaching into higher altitude regions in Ecuador, currently reaching 1,541 m there [6]. The fact that MaxEnt projected a near-present maximum elevation of 1,937 m and that the E_max_ has been reported to reach 1,941 m in the M-C region [3] provides additional confidence in our MaxEnt results. Within the M-C region, approximately 354,000 km^2^ (or about 9.6% of the study area) is 2,000 m or greater in elevation, with most of this high-elevation terrain in Mexico, Guatemala, Costa Rica, and the northern Cordilleras of Andes in Colombia and Venezuela. It is worth noting that approximately 47 million people live in areas of 2,000 m or greater in these countries [20], and many more live in potentially high-risk areas in the Andean region to the south. Therefore, the approach we used here with MaxEnt may be applicable to many other areas where anophelines are likely to colonize high-elevation environments, such as East Africa [21].

The fact that MaxEnt projected near-present occurrence of the vector in Trinidad and other portions of the southern Caribbean islands where it is generally absent [5] suggests that the outputs from our experiments represent potential as well as actual distribution. In this sense, relatively high p(***s***) values obtained from climate-based MaxEnt experiments should be interpreted with some caution as the probability surfaces may be better construed as areas where the vector is likely to find suitable habitat if a population becomes established. Given the movement of people and goods within the region and the transportation connections between countries and islands, it is possible to imagine many potential *An. albimanus* invasions of insular locations from nearby mainland environments. However, such factors as competitive exclusion, existing vector control measures and variable socio-economic linkages between sources and sinks (i.e., potentially suitable areas for colonization) may limit the chance establishment of breeding populations. Indeed, socio-economic, land cover, and human population can be factored into future experiments. However, in this study, we constrained our analysis to climate and topographic covariates because these are relatively static over decadal time scales and we could design model experiments so that they were directly comparable between the near-present and 2080 conditions. Owing to the relatively coarse 2.5 arc-minute resolution of our data, we did not explicitly examine interactions between slope, aspect and rainfall variability, which can be readily discerned at finer resolutions and at more local scales of analysis. However, future work conducted at higher spatial resolutions and at national-to-subnational scales may benefit from use of higher resolution data as well as incorporation of topographic wetness indices, which have provided meaningful predictors of anopheline presence in other studies [10]. Further, it may be possible to use MaxEnt and other SDMs with future human population and land cover projections in such a way as to reduce any “false alarms” or commission errors such as the one noted for Trinidad. In addition, among different SDMs, MaxEnt is potentially better at spatial interpolation than extrapolation, so other SDMs such as GARP may be better suited for projecting future latitudinal expansion of the vector’s range [12]. For example, Townsend Peterson [22] used GARP to project future distribution of the *An. gambiae* complex and *An. arabiensis* in Africa and he demonstrated that model’s utility for projecting future distribution of these vectors. Interestingly, this study also suggested that future climate scenarios will result in net declines in the areas exposed to these important vector species.

## Conclusions

Our climate-based investigation adds to the small group of published studies [12,13,22] that have used SDMs to project the ranges of anopheline mosquitoes and is among the first to use SDMs with different GCM outputs to project future ranges. We suggest that the results have the potential to inform current and future integrated control strategies, at broad scales, and are particularly applicable to further investigation of how elevation-climate linkages may control the spread of anopheline vectors of human malaria.

## Methods

### Model

MaxEnt is a popular and robust species distribution model or SDM [10,23,24]. We used this model to generate probability surfaces that depict the probability of presence, p(***s***), of *An. albimanus* in Mesoamerica and the Caribbean (Figure 1) from climate and topographic data. We selected MaxEnt because it produces accuracies that typically compare with or exceed other SDMs [13,24,25]. MaxEnt uses a maximum entropy approach that integrates model covariate selection and controls for overfitting by using smoothing and identifies how the covariates (i.e., spatial layers representing environmental variables or **z**) contribute to the model [23,24]. In fact, the model minimizes relative entropy, a measure of dispersion or uncertainty associated with a random variable, through a Gibbs distribution, which is an exponential family model:

(1) $$f_{1}\left(z\right)=f\left(z\right){\text{e}}^{n\left(z\right)}$$

where ***f***_1_(**z**) is the probability density of covariates across a landscape at known species locations and ƞ(**z**) = α + β·h(**z**). α is a normalizing constant that ensures ***f***_1_(**z**) sums to 1 and β is an estimated parameter that weighs the contribution of each covariate using a log likelihood approach [24]. For a complete explanation of MaxEnt, readers are referred to Elith et al. [24].

### Mosquito presence data

We utilized three sources of presence data including points included in The Global Diversity Information Facility (http://www.gbif.org), MosquitoMap (http://www.mosquitomap.org) and published data from mosquito surveys in Colombia [4]. The presence data included only collection records from 1950–2000 to coincide with the time period represented by the near-present climate layers. We gridded presence points to 2.5 arc-minute spatial resolution, which yielded 350 unique *An. albimanus* pixels throughout Caribbean Basin and northern South America, a sample size sufficient to model the species’ potential distribution based on environmental conditions at locations of known occurrence [26].

### Environmental data

**Figure 5:**
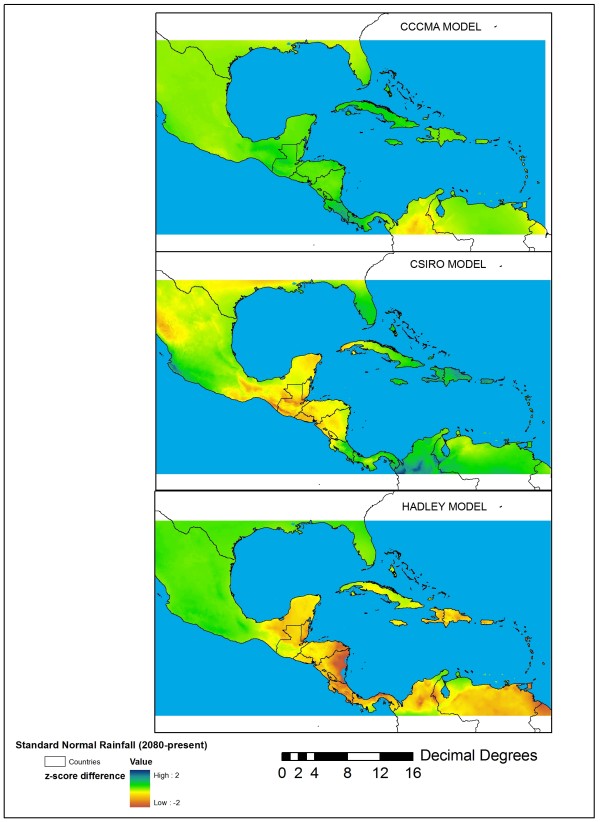
Differences in maximum temperature (°C × 10) between mean-monthly temperature from near-present climate and mean monthly temperature for 2080 obtained from the three GCMs used in this study: CCCMA (top panel), CSIRO (middle panel), and the Hadley Centre model (bottom panel).

**Figure 6:**
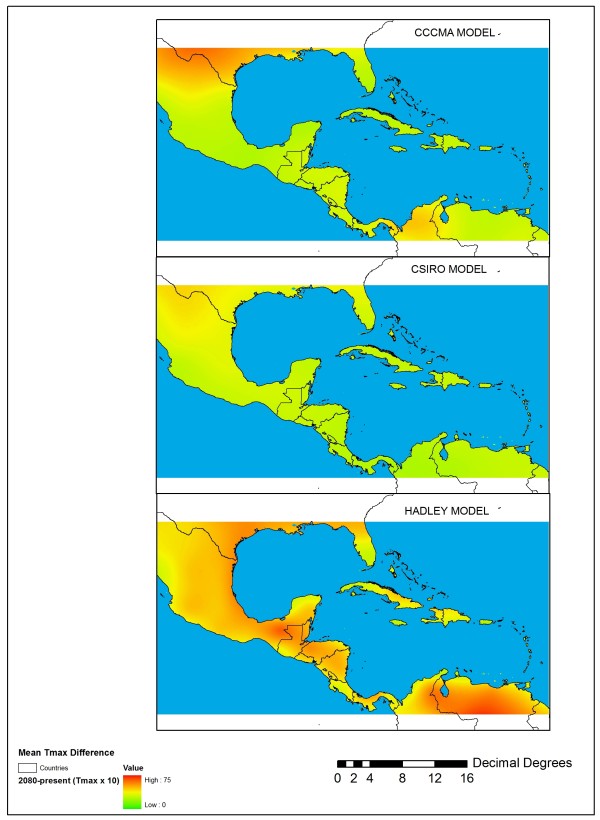
Differences in total annual rainfall amounts, normalized using a Z-scores, between near-present climate and 2080 climates obtained from obtained from the three GCMs used in this study: CCCMA (top panel), CSIRO (middle panel), and the Hadley Centre model (bottom panel).

We utilized climate layers from the WorldClim database (http://www.worldclim.org) gridded to 2.5 arc-minute (~8 km) resolution and topography data from derived from the Shuttle Radar Topography Mission (available through the Global Land Cover Facility, http://glcf.umiacs.umd.edu) as covariates to estimate the present distribution of *An*. *albimanus*. We also modeled the future 2080 distribution of *An. albimanus* using three different general circulation models (GCMs) that project future changes in temperature and precipitation patterns based on the A2 socio-economic scenario, which assumes globally convergent total fertility rates, growth-oriented economies with regional variation, and is considered to be a more pessimistic socio-economic outcome resulting in continued growth in emissions of warming gases [14]. Thus, the A2 family of scenarios represents a potential worst case in terms of future climate change. The specific GCM outputs selected were from widely used GCMs developed by the Canadian Centre on Climate Modelling and Analysis (CCCMA), the Australian Commonwealth Scientific and Industrial Research Organization (CSIRO) and the Hadley Centre (HadCM3) obtained from http://www.worldclim.org/futdown.htm. Each model produces different temperature and rainfall predictions for our study region and therefore represents a potential range of outcomes by 2080. We selected these three GCMs because they produce very different predictions for future rainfall and temperature in the M-C region (Figures 5 and 6). In particular, the Hadley Center model produced the largest departures in terms of future mean monthly temperature maximum and rainfall, which is given as the normalized (z-score) difference between mean annual rainfall in 2080 and mean annual rainfall in the 1950–2000 period.

### Model experiments and threshold selection

We conducted 70 different MaxEnt experiments using different combination of monthly minimum and maximum temperature, monthly precipitation and SRTM layers. An experiment was defined as a unique set of environmental covariates in which different combinations of temperature, rainfall, elevation, and bias layers were input to the model. Each set of twelve monthly T_max_, T_min_, and rainfall surfaces from the WorldClim data was treated as single covariate to include the full seasonal influence of these climate drivers (Table 1). Although MaxEnt can accept an unlimited number of covariates, including nominally scaled variables such as land cover, we restricted model experiments to covariates that are either expected to remain static, such as topography, or sets of climate covariates that are directly comparable between near-present and 2080 conditions. Of the total 350 unique presence points, we randomly selected 36 presence points to independently validate the results of each model experiment. These independent testing points (Figure 1) were used to calculate statistics such as the mean p(***s***) and standard deviation for each model experiment as well as commission and omission errors to evaluate the accuracy of different experiments. We developed several different bias layers intended to de-bias the data for unrepresentative (i.e., highly clustered) sampling of specimens. After a number of trials with different bias layers, we utilized a bias layer based on examination of the point distribution in Figure 1, which shows a clear tendency for collections near coastlines, as well as informed assumptions based on our own field survey experience; i.e., that the probability of sampling for *An. albimanus* declines in a negative exponential (i.e., following a decay function) fashion as a function of distance from major roads and coasts. To create this particular bias layer, we used GIS software to generate distance-weighted fuzzy membership layers, scaled from 0–255, with a negative J-shaped function fuzzy function and breakpoints at 5 km for both roads and coastlines. The two fuzzy layers were then combined using a weighted linear combination that assigned equal weights to each layer. We found that this approach consistently improved the mean p(***s***) values obtained from the validation points relative to other approaches (e.g., fuzzy layers based on distance from rivers, or distance from roads alone). Thus, each experiment was evaluated both qualitatively by comparing the MaxEnt outputs with literature reports from different countries and parts of the region and quantitatively by extracting probability values using the independent validation points selected randomly from the data set.

For further analysis, we selected three model experiments for the both near-present and 2080 projections based on their relatively high mean values (i.e., ~0.70) from the independent test points and to illustrate model sensitivity with inclusion/exclusion of different covariates. We then ran the model with the 2080 climate layers and the SRTM layer to produce future probability surfaces using the same combination of covariates used in experiment 61. We used the lowest presence threshold (LPT) method for setting thresholds to evaluate presence/absence from the MaxEnt experiments. This method uses the lowest predicted value associated with any one of the observed presence points and it can be interpreted ecologically as pixels predicted as being at least as suitable as those where a species’ presence has been recorded [27]. It is thus considered a highly conservative way to map the minimum predicted distribution. In addition, the LPT approach reduces omission error to zero in the training data set [27]. LPT-based maps were then cross-tabulated for near-present and 2080 conditions to indicate areas of model agreement and disagreement. We also applied a logical threshold of p(***s***) > 0.50 after examining mean values for validation points. We combined different presence/absence maps by cross-tabulating the maps thresholded with the LPT criterion and retaining the area where all three models agreed. This approach of combining different maps provides a conservative estimate of near-present and 2080 model experiments and reduces further the likelihood of false alarms (commission errors).

## Competing interests

The authors declare that they have no competing interests.

## Authors’ contributions

DOF, MLAF, MLQ, SH, and JCB conceived the study and wrote the paper. DOF conducted the MaxEnt experiments and model validation. MLAF and MLQ provided presence points from Colombia for *An. albimanus*. All authors have approved the final manuscript.
